# Local Transmission of SARS-CoV-2 Lineage B.1.1.7, Brazil, December 2020

**DOI:** 10.3201/eid2703.210038

**Published:** 2021-03

**Authors:** Ingra Morales Claro, Flavia Cristina da Silva Sales, Mariana Severo Ramundo, Darlan S. Candido, Camila A.M. Silva, Jaqueline Goes de Jesus, Erika R. Manuli, Cristina Mendes de Oliveira, Luciano Scarpelli, Gustavo Campana, Oliver G. Pybus, Ester Cerdeira Sabino, Nuno Rodrigues Faria, José Eduardo Levi

**Affiliations:** University of São Paulo, São Paulo, Brazil (I.M. Claro, F.C.S. Sales, M.S. Ramundo, D.S. Candido, C.A.M. Silva, J.G. de Jesus, E.R. Manuli, E.C. Sabino, N.R. Faria, J.E. Levi);; University of Oxford, Oxford, UK (D.S. Candido, O.G. Pybus, N.R. Faria);; Diagnósticos da América SA (DASA), Baueri, Brazil (C.M. de Oliveira, L. Scarpelli, G. Campana, J.E. Levi);; Imperial College London, London, UK (N.R. Faria)

**Keywords:** 2019 novel coronavirus disease, coronavirus disease, COVID-19, severe acute respiratory syndrome coronavirus 2, SARS-CoV-2, viruses, respiratory infections, zoonoses, transmission, genomic, surveillance, lineage B.1.1.7, Brazil

## Abstract

In December 2020, research surveillance detected the B.1.1.7 lineage of severe acute respiratory syndrome coronavirus 2 in São Paulo, Brazil. Rapid genomic sequencing and phylogenetic analysis revealed 2 distinct introductions of the lineage. One patient reported no international travel. There may be more infections with this lineage in Brazil than reported.

Genomic sequencing and analysis during the severe acute respiratory syndrome coronavirus 2 (SARS-CoV-2) pandemic have led to identification of ≈800 distinct SARS-CoV-2 lineages worldwide. A new phylogenetic cluster, B.1.1.7 lineage or variant of concern 202012/01, is characterized by 17 unique mutations and was first detected in southeastern England in late September 2020 (A. Rambaut et al., unpub. data, https://virological.org/t/preliminary-genomic-characterisation-of-an-emergent-sars-cov-2-lineage-in-the-uk-defined-by-a-novel-set-of-spike-mutations/563). As of January 17, 2021, this lineage had been confirmed in 38 countries (https://cov-lineages.org/global_report_B.1.1.7.html). Epidemiologic and phylogenetic studies suggest that the rapid epidemic growth of B.1.1.7 in the United Kingdom is caused by its higher transmissibility (E. Volz et al., unpub. data, https://www.medrxiv.org/content/10.1101/2020.12.30.20249034v2; N. Davies, unpub. data, https://cmmid.github.io/topics/covid19/uk-novel-variant.html), which could lead to increased incidence and higher peaks in hospitalizations and deaths (N. Davies, unpub. data, https://cmmid.github.io/topics/covid19/uk-novel-variant.html).

We confirm 2 cases of infection with SARS-CoV-2 B.1.1.7 lineage in Latin America. On December 30, 2020, we received saliva samples from 2 patients for genomic sequencing as part of research surveillance activities. Patient 1 was a woman 20–30 years of age residing in São Paulo, Brazil, who reported no travel outside of Brazil. Her symptoms began on December 21, and testing was conducted the next day. Patient 2 was a man 30–40 years of age who was tested in São Paulo on December 22 after having traveled from London on December 19. Ethics approval for this study was confirmed by the national ethics review board (Comissão Nacional de Ética em Pesquisa, protocol no. CAAE 30127020.0.0000.0068). 

PCR testing (TaqPath COVID-19 PCR; ThermoFisher Scientific, https://www.thermofisher.com) performed as previously described (*1*) indicated that patient 1 was positive for the open reading frame 1ab (cycle threshold [C_t_] 25.8) and nucleoprotein (C_t_ 24.5) gene targets and patient 2 was positive for open reading frame 1ab (C_t_ 28.1) and nucleoprotein (C_t_ 27.29), but both were negative for the spike gene target. The 2 spike-gene dropout samples were identified among 400 samples collected during November 4–December 25, 2020. 

For each sample, we conducted nanopore sequencing in duplicate by using the ARTIC protocol (https://www.protocols.io/view/ncov-2019-sequencing-protocol-bbmuik6w). Concentrations of double-stranded DNA for the library-negative controls were below detection levels, indicating no contamination. We conducted whole-genome sequencing of SARS-CoV-2 by using the MinION platform (Oxford Nanopore Technologies, https://nanoporetech.com). By December 31, sequencing statistics revealed 56,565 mapped reads for patient 1 and 51,761 for patient 2. Consequently, 28,023 bases for patient 1 and 26,339 for patient 2 were covered at >25× depth. Consensus sequences covered 92.4% of the Wuhan Hu-1 reference genome (GenBank accession no. MN908947.3) for patient 1 and 87.1% for patient 2. For the 2 newly generated genome sequences, we identified the B.1.1.7 lineage (assignment probability = 1.0) by using the pangolin COVID-19 Lineage Assigner version 2.1.6 (*2*) (https://pangolin.cog-uk.io). The 2 genomes were made publicly available on GISAID (http://www.gisaid.org) on December 31, 2020 (identification nos. EPI_ISL_754236 for patient 1 and EPI_ISL_754237 for patient 2).

We next estimated a rapid maximum-likelihood phylogenetic tree (*3**,**4*) for a multiple sequence alignment (*5*) with the new sequences and 127 publicly available B.1.1.7 genomes from around the world available on GISAID (*6*) as of December 31, 2020 (https://github.com/CADDE-CENTRE/VOC-Lineage-Brazil). The virus genome recovered from patient 1 grouped within a well-supported cluster (bootstrap 85%) of 10 sequences (60% from the United Kingdom) (Figure). This finding is consistent with the travel history of an asymptomatic family member who was positive for SARS-CoV-2 (according to a rapid test performed on December 23, 2020), who arrived in São Paulo on December 17 after traveling from Italy to the United Kingdom and, after a short stay, from London to São Paulo, and who was in close contact with patient 1. The sequence from patient 2 clustered with good statistical support (bootstrap 79.4%) with a sequence collected in the United Kingdom on November 27. Patient 2 had traveled from London to São Paulo on December 19 and was symptomatic when saliva was collected on December 22. Phylogenetic analysis suggests that this infection represents a second, independent introduction of the B.1.1.7 lineage from the United Kingdom to Brazil; patient 2 was not epidemiologically linked to patient 1.

**Figure:**
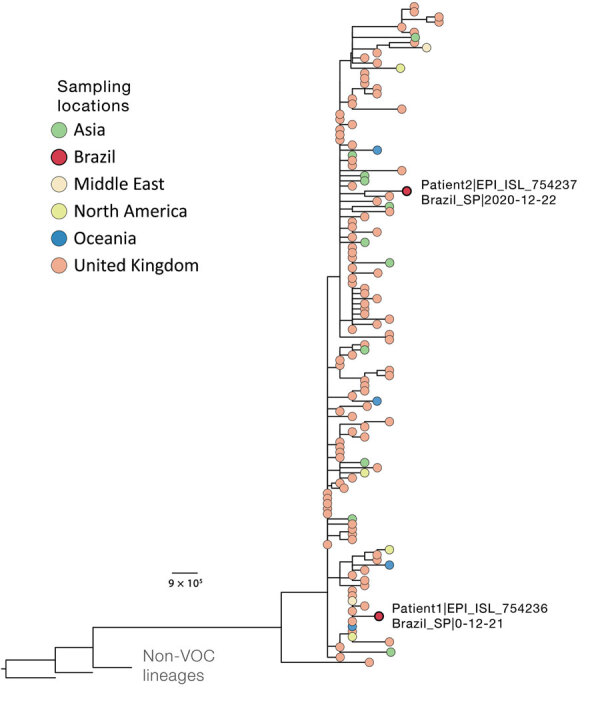
Phylogenetic context of novel severe acute respiratory syndrome coronavirus 2 B.1.1.7 genomes isolated from 2 patients in Brazil (labeled on figure), December 2020. Downsampling for the phylogenetic analysis of the B.1.1.7 SARS-CoV-2 variant (n = 4,693, December 31, 2020) was performed by selecting 1 sequence per country per day. As outgroups, we included 2 B.1.1 sequences from the United Kingdom that were closely related to the lineage of interest and sequence WH04 from Wuhan, China (GISAID identification no. EPI_ISL_406801; http://www.gisaid.org). Details on multiple alignment and phylogenetic tree reconstruction are described elsewhere (*4*). Tree file, aligned sequences, and GISAID acknowledgment tables are available at https://github.com/CADDE-CENTRE/VOC-Lineage-Brazil. Scale bar indicates nucleotide substitutions per site. VOC, variant of concern.

Because information about this lineage from locations outside the United Kingdom is limited, our interpretations based on phylogenetic data might be biased by the different numbers of available genome sequences shared around the globe. Moreover, the samples that we analyzed were selected from only 2 cases confirmed by reverse transcription PCR in São Paulo; thus, our genomes were obtained from a small fraction of targeted spike-gene failure, and frequency of detection in our nonrandom sample does not represent prevalence of this lineage at the population level.

Despite temporary suspension of all flights to or from Brazil from or through the United Kingdom as of December 25, 2020 (https://www.gov.uk/foreign-travel-advice/brazil), it is likely that the number of SARS-CoV-2 lineage B.1.1.7 infections in Brazil is higher than that reported. Increasing genomic surveillance of B.1.1.7 and other variants of concern that carry mutations of potential biological significance (e.g., E484K in the spike protein; C.M. Voloch, unpub data, https://www.medrxiv.org/content/10.1101/2020.12.23.20248598v1) is imperative for monitoring vaccination effectiveness and contextualizing the epidemiology and evolution of SARS-CoV-2 in Latin America.
